# Telemedicine for cancer patients during COVID-19 pandemic: between threats and opportunities

**DOI:** 10.2217/fon-2020-0324

**Published:** 2020-05-01

**Authors:** Ronaldo Elkaddoum, Fady Gh Haddad, Roland Eid, Hampig Raphael Kourie

**Affiliations:** 1 Department of Hematology-Oncology, Faculty of Medicine, Saint Joseph University of Beirut, Beirut, Lebanon

**Keywords:** COVID-19, telehealth, telemedicine, teleoncology

SARS-CoV-2 or COVID-19 is a novel strain of coronavirus. To this date (the 7th of April 2020), around 1.4 million individuals have been affected by the disease worldwide, with nearly 80,000 deaths, according to the WHO [1]. In the general population, the vast majority of forms of the disease are benign, with mild to moderate respiratory symptoms. However, patients who are immunosuppressed or with underlying comorbidities, like cancer patients, are at a higher risk of developing more serious complications, such as more frequent pneumonias, a higher rate of hospitalization, respiratory failure or multiple organ failure and even death [2]. Moreover, governments are imposing confinement rules, limiting all kinds of transportation. This has led to the reliance on telecommunications, from online education and remote working from home, to telemedicine. Many medical societies and physicians are adopting this option as the safest, both for the patient and the medical staff [3]. This new perspective has led us to wonder whether telemedicine is a threat to oncology patient care or an opportunity that could revolutionize our clinical practice.

When we talk about telemedicine, as with other technologies, the first concern is always its accessibility. Although establishing contacts is not difficult, a stable connection in remote areas is often problematic. The concern grows bigger for poorer patients with limited access to the internet. Using the same sociological approach, patients may simply refuse to receive care from a distance, while others may insist on this technology when it may be necessary for them to go to the clinic. Another obstacle could be the impossibility of carrying out an appropriate clinical examination from a distance, knowing that this routine clinical practice often reveals early signs which could lead to new investigations. The biggest problem of all might be the selection of patients who are asked to come to the clinic and those who will be followed at home. Patients can be divided into three subgroups with different recommendations: cancer patients in the follow-up phase or on oral therapy; patients with a recently diagnosed cancer under treatment with curative intent; and metastatic cancer patients receiving palliative treatment [4]. The European Society of Medical Oncology issued guidelines [5] concerning patient care during the pandemic. In breast cancer management [6] for example, it was clearly recommended to switch to telemedicine as much as possible for patients who present new symptoms or side effects, despite being considered high-to-medium priority patients. The recommendations are necessary for practitioners, but good triage is based on the physician’s experience alongside the apparent state and performance status of the patient, that could sometimes be misleading. Finally, we approach the psychological aspect of the fight against cancer, where keeping the patient’s will is of the utmost importance. The lack of direct contact between the patient and the oncologist can adversely affect the patient’s well-being, motivation and sense of security, especially in times of uncertainty such as the current pandemic period. To tackle the issue European Society of Medical Oncology recommended the use of a web support [7], which can alleviate the problem to a big extent.

Nevertheless, tele-oncology has many advantages and its large-scale practice could change a lot in the approach of our daily practice when everything goes back in order. Concerning the battle against COVID-19, it helps reducing the demands on the personal protective equipment (PPE). PPE consists of medical masks, respirator N95 or FFP2 standard, gloves, gowns and eye protection by goggles or face shield. However, the demand is increasing worldwide due to the surge of COVID-19 cases but also to massive panic buying and stockpiling among populations. Combined with the incapacity to meet the rising demands on PPE, this will lead to further PPE stock shortages. When using telemedicine, healthcare workers could follow-up on patients, discuss their results, evaluate their response to therapy and also asses patients suspected of COVID-19 infection. This approach minimizes the need for individuals to visit healthcare facilities, leading to a lesser consumption of PPE by the patients and the doctors, thus saving it for other healthcare professionals and for indicated situations PPE [8].

Starting with the fact that some patients living in remote areas, who usually need to travel a long and tiring road, can now easily address their physician for routine questions and answers or updates from the comfort of their bed. They will not have to go through the long hours of waiting in clinics and above all will not be exposed to other patients who could be carriers of pathogens, from COVID-19 to the most common infectious agents. This is particularly important for patients who have to come by in order to refill prescriptions, get test results and analysis or be monitored when they take oral therapy. Another advantage is that the clinician can easily and more frequently ensure that the patients are adhering to the given recommendations, by sending them checklists or forms to fill at a daily basis. In addition, patients can more easily address their physician with new symptoms, fears or questions. On another note, tele-oncology can be seen as an opportunity to develop peripheral care centers, which would follow the latest recommendations, ensuring regular and on-demand teleconferences with specialists working in specialized tertiary medical. Finally, this can facilitate communication between different specialists, favoring a multidisciplinary approach in patient care. A final essential point to tackle is the confidentiality of information, which is our ethical duty to protect against leaks, using trusted end-to-end encrypted text applications. For example, the French government published a review on the applications that can be used during the COVID-19 crisis for telemedicine in general, including security review and payment options [9]. In the US, some specialized cancer centers are encouraging the use of apps such as MyChart to facilitate cancer patient care [10]. Moreover, in Lebanon, the National Social Security Fund, one of the biggest third-party payers is now giving on the spot online confirmations for payments thus making the access for healthcare easier for cancer patients. Big tertiary care centers are giving video consultations online via applications such as Cisco WebEx or Teams and even coordinating online with the National Social Security Fund.

Telehealth in oncology with it advantages and setbacks is nothing new [11]. It has been already here for years and used especially to connect with rural areas with satisfying results [12]. With the emergence of COVID-19, cities lockdown and fear from contagion, the advantages of teleoncology are clearly outweighing the setbacks. This is making the use of such a technology widely accepted in both physicians and patients’ mediums. Since with growing demand comes an increase in offer, many developers have published new applications, other have improved already existing ones. What is more important is that both oncologists and patients are learning to use these technologies. With that being said, there is certainly a need for large scale studies in order to see how to improve telehealth and if it will be very soon a new standard of care in oncology.

## Conclusion

Although the first priority today is to limit the spread of COVID-19 along with its death toll, we can see that this gives us an excellent opportunity to improve accessibility to oncology care and by paying attention to the possible setbacks, it can open a window for new practices that are more inclusive for the coming decade.
